# 4-Bromo­meth­yl-1-phenyl­sulfon­yl-1*H*-indole

**DOI:** 10.1107/S1600536808007794

**Published:** 2008-03-29

**Authors:** G. Chakkaravarthi, Radhakrishnan Sureshbabu, A. K. Mohanakrishnan, V. Manivannan

**Affiliations:** a Department of Physics, CPCL Polytechnic College, Chennai 600 068, India; b Department of Organic Chemistry, University of Madras, Guindy Campus, Chennai 600 025, India; c Department of Physics, Presidency College, Chennai 600 005, India

## Abstract

In the title mol­ecule, C_15_H_12_BrNO_2_S, the indole mean plane and phenyl ring are nearly orthogonal to each other, forming a dihedral angle of 88.19 (13)°. The Br atom is disordered over two close positions with occupancies of 0.56 (4) and 0.44 (4). The crystal packing exhibits weak inter­molecular C—H⋯π inter­actions.

## Related literature

For related crystal structures, see: Chakkaravarthi *et al.* (2007 ▶, 2008 ▶). For biological activities of indole derivatives, see: Okabe & Adachi (1998 ▶); Schollmeyer *et al.* (1995 ▶).
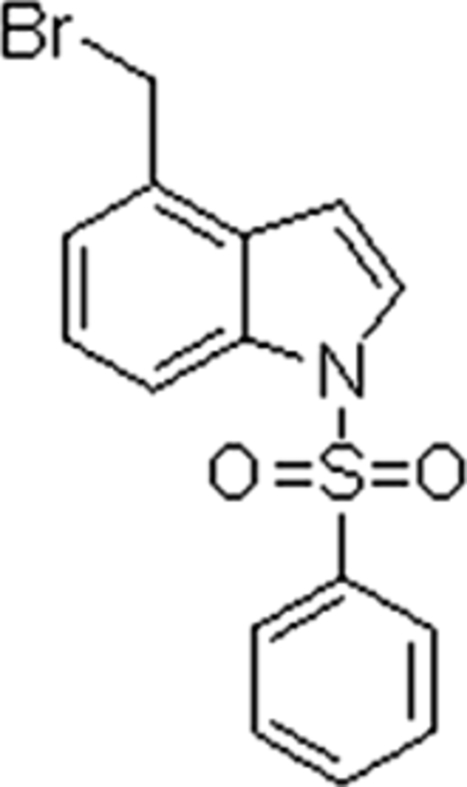


## Experimental

### 

#### Crystal data

C_15_H_12_BrNO_2_S
*M* *_r_* = 350.23
Monoclinic, 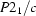

*a* = 11.7060 (9) Å
*b* = 8.2399 (7) Å
*c* = 15.4495 (11) Å
β = 103.858 (3)°
*V* = 1446.8 (2) Å^3^
*Z* = 4
Mo *K*α radiation
μ = 2.98 mm^−1^
*T* = 295 (2) K
0.18 × 0.18 × 0.16 mm

#### Data collection

Bruker Kappa APEX2 diffractometer
Absorption correction: multi-scan (*SADABS*; Sheldrick, 1996 ▶) *T*_min_ = 0.566, *T*_max_ = 0.620
16472 measured reflections
3606 independent reflections
2160 reflections with *I* > 2s(*I*)
*R*_int_ = 0.041

#### Refinement

*R*[*F* ^2^ > 2σ(*F* ^2^)] = 0.062
*wR*(*F* ^2^) = 0.199
*S* = 1.06
3606 reflections
191 parameters
8 restraints
H-atom parameters constrained
Δρ_max_ = 1.25 e Å^−3^
Δρ_min_ = −0.54 e Å^−3^

### 

Data collection: *APEX2* (Bruker, 2004 ▶); cell refinement: *APEX2*; data reduction: *APEX2* program(s) used to solve structure: *SHELXS97* (Sheldrick, 2008 ▶); program(s) used to refine structure: *SHELXL97* (Sheldrick, 2008 ▶); molecular graphics: *PLATON* (Spek, 2003 ▶); software used to prepare material for publication: *SHELXL97*.

## Supplementary Material

Crystal structure: contains datablocks I, global. DOI: 10.1107/S1600536808007794/cv2392sup1.cif

Structure factors: contains datablocks I. DOI: 10.1107/S1600536808007794/cv2392Isup2.hkl

Additional supplementary materials:  crystallographic information; 3D view; checkCIF report

## Figures and Tables

**Table 1:** Hydrogen-bond geometry (Å, °)

*D*—H⋯*A*	*D*—H	H⋯*A*	*D*⋯*A*	*D*—H⋯*A*
C13—H13⋯*Cg*1^i^	0.93	2.83	3.716 (6)	160
C9—H9*D*⋯*Cg*1^ii^	0.97	2.92	3.673 (5)	135
C1—H1⋯*Cg*2^iii^	0.93	2.69	3.584 (6)	162

Symmetry codes: (i) 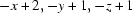; (ii) 
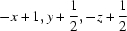
; (iii) 
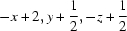
. *Cg*1 and *Cg*2 are the centroids of atoms C3–C8 and C10–C15, respectively.

**Fig. 1.**
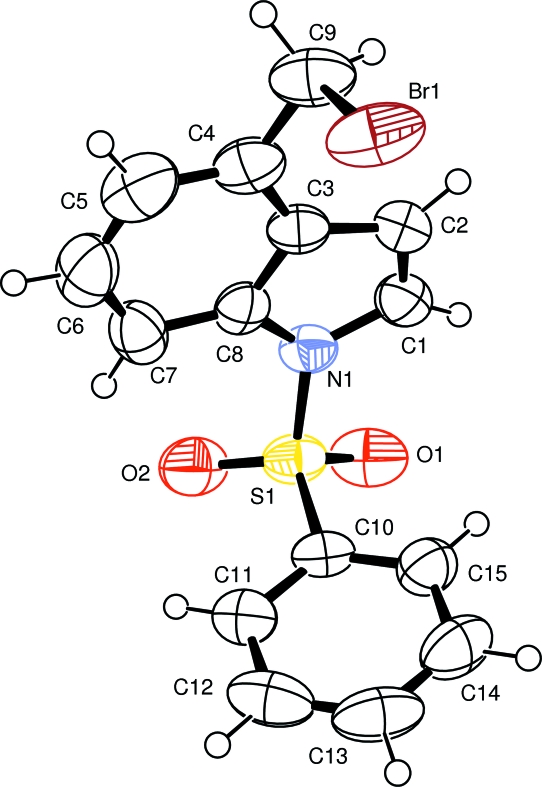
The molecular structure of (I) showing the atomic labels and 50% probability displacement ellipsoids for non-H atoms. Only major parts of the disordered atoms are drawn.

## supplementary crystallographic information

### Comment

The indole derivatives are found to possess antibacterial (Okabe and Adachi, 1998) and antitumour (Schollmeyer *et al.*, 1995) activities. In continuation of our studies in indole derivatives, we present the crystal structure of the title compound (I).

The geometric parameters of (I) (Fig. 1) agree with those in the reported structures (Chakkaravarthi *et al.*, 2007; 2008) The indole mean plane and phenyl ring are nearly orthogonal to each other forming a dihedral angle of 88.19 (13)°. The plane of N1/S1/C1 makes the dihedral angles of 84.30 (14)°) and 72.38 (16)°, respectively, with the indole mean plane and phenyl ring. The sum of bond angles around N1 (356.9°) indicates that N1 is *sp*^2^-hybridized. The torsion angles C11-C10-S1-O2 [-6.3 (5)°] and C15-C10-S1-O1 [39.4 (5)°] indicate the *syn* conformation of the sulfonyl moiety.

The crystal packing exhibits weak intermolecular C—H···π interactions, involving the rings C3-C8 (centroid *Cg*1) and C10-C15 (centroid *Cg*2) (Table 1).

### Experimental

4-(Methyl)-1-(phenylsulfonyl)-1*H*-indole (1 g, 2.8 m.mol), *N*-bromo succinimide (0.5 g, 3 m.mol), azobis isobutyro nitrile (50 mg) were dissolved in 50 ml of carbon tetra chloride and refluxed on a waterbath for 2 h, then cooled to the room temperature. Succinimide was filtered off over sodium sulfate. Filtrate was evaporated under reduced pressure. Product was recrystallized from methanol. Yield: 80 %.

### Refinement

H atoms were positioned geometrically and refined using riding model with C-H = 0.93-0.97 Å, and *U*_iso_(H) = 1.2Ueq(C). The Br atom was treated as disordered over two close positions with the occupancies of 0.56 (4) and 0.44 (4), respectively. The distances C4-C5, C10-C11, C11-C12, C12-C13, C13-C14, C14-C15, C15-C10 were restrained to 1.395 (5) Å and the distance C9-Br1A was restrained to 1.91 (10) Å. The positive residual peak 1.26 e Å^-3^ is located at  1.62 Å from C9; the peak might be the disordered component of Br with small occupancy. It was ignored as showing no any structural or packing consequences.

### Crystal data

**Table:**

C_15_H_12_BrNO_2_S	*F*_000_ = 704
*M**_r_* = 350.23	*D*_x_ = 1.608 Mg m^−^^3^
Monoclinic, *P*2_1_/*c*	Mo *K*α radiation λ = 0.71073 Å
Hall symbol: -P 2ybc	Cell parameters from 5113 reflections
*a* = 11.7060 (9) Å	θ = 2.5–25.8º
*b* = 8.2399 (7) Å	µ = 2.99 mm^−^^1^
*c* = 15.4495 (11) Å	*T* = 295 (2) K
β = 103.858 (3)º	Block, colourless
*V* = 1446.8 (2) Å^3^	0.18 × 0.18 × 0.16 mm
*Z* = 4	

### Data collection

**Table:**

Bruker Kappa APEX2 diffractometer	3606 independent reflections
Radiation source: fine-focus sealed tube	2160 reflections with *I* > 2s(*I*)
Monochromator: graphite	*R*_int_ = 0.041
*T* = 295(2) K	θ_max_ = 28.6º
ω and φ scan	θ_min_ = 1.8º
Absorption correction: multi-scan(SADABS; Sheldrick, 1996)	*h* = −15→15
*T*_min_ = 0.566, *T*_max_ = 0.620	*k* = −10→11
16472 measured reflections	*l* = −12→20

### Refinement

**Table:**

Refinement on *F*^2^	Secondary atom site location: difference Fourier map
Least-squares matrix: full	Hydrogen site location: inferred from neighbouring sites
*R*[*F*^2^ > 2σ(*F*^2^)] = 0.062	H-atom parameters constrained
*wR*(*F*^2^) = 0.199	*w* = 1/[σ^2^(*F*_o_^2^) + (0.089*P*)^2^ + 1.9138*P*] where *P* = (*F*_o_^2^ + 2*F*_c_^2^)/3
*S* = 1.06	(Δ/σ)_max_ < 0.001
3606 reflections	Δρ_max_ = 1.25 e Å^−^^3^
191 parameters	Δρ_min_ = −0.54 e Å^−^^3^
8 restraints	Extinction correction: none
Primary atom site location: structure-invariant direct methods	

### Fractional atomic coordinates and isotropic or equivalent isotropic displacement parameters (Å^2^)

**Table:**

	*x*	*y*	*z*	*U*_iso_*/*U*_eq_	Occ. (<1)
C1	0.7826 (5)	0.6808 (6)	0.2167 (3)	0.0595 (13)	
H1	0.8345	0.7022	0.1810	0.071*	
C2	0.7274 (4)	0.7938 (6)	0.2531 (3)	0.0576 (12)	
H2	0.7326	0.9055	0.2463	0.069*	
C3	0.6578 (4)	0.7104 (6)	0.3052 (3)	0.0501 (11)	
C4	0.5821 (4)	0.7671 (7)	0.3555 (3)	0.0605 (13)	
C5	0.5258 (5)	0.6520 (8)	0.3953 (4)	0.0771 (18)	
H5	0.4734	0.6856	0.4284	0.092*	
C6	0.5457 (6)	0.4882 (9)	0.3870 (4)	0.0769 (18)	
H6	0.5063	0.4147	0.4151	0.092*	
C7	0.6223 (5)	0.4290 (7)	0.3385 (4)	0.0643 (14)	
H7	0.6367	0.3186	0.3345	0.077*	
C8	0.6757 (4)	0.5440 (6)	0.2967 (3)	0.0489 (11)	
C10	0.9404 (4)	0.3588 (5)	0.3317 (3)	0.0502 (11)	
C11	0.9363 (5)	0.2442 (6)	0.3965 (3)	0.0673 (14)	
H11	0.8754	0.1690	0.3884	0.081*	
C12	1.0253 (5)	0.2450 (8)	0.4736 (3)	0.0833 (19)	
H12	1.0248	0.1697	0.5183	0.100*	
C13	1.1153 (5)	0.3580 (7)	0.4842 (4)	0.0805 (19)	
H13	1.1751	0.3569	0.5361	0.097*	
C14	1.1177 (5)	0.4719 (7)	0.4194 (3)	0.0765 (17)	
H14	1.1783	0.5477	0.4277	0.092*	
C15	1.0293 (4)	0.4726 (6)	0.3419 (3)	0.0621 (13)	
H15	1.0297	0.5484	0.2973	0.075*	
N1	0.7516 (3)	0.5255 (5)	0.2396 (3)	0.0524 (10)	
O1	0.8833 (4)	0.3941 (6)	0.1596 (2)	0.0771 (12)	
O2	0.7575 (3)	0.2269 (5)	0.2295 (3)	0.0775 (11)	
S1	0.83141 (11)	0.36176 (16)	0.23203 (8)	0.0569 (4)	
C9	0.5669 (4)	0.9457 (7)	0.3700 (3)	0.0725 (16)	
H9A	0.4865	0.9665	0.3734	0.087*	0.56 (4)
H9B	0.5813	1.0060	0.3198	0.087*	0.56 (4)
H9C	0.4898	0.9633	0.3813	0.087*	0.44 (4)
H9D	0.5701	1.0035	0.3160	0.087*	0.44 (4)
Br1	0.6721 (4)	1.0180 (7)	0.4768 (3)	0.0792 (10)	0.56 (4)
Br1A	0.6841 (7)	1.0268 (5)	0.4688 (5)	0.0844 (16)	0.44 (4)

### Atomic displacement parameters (Å^2^)

**Table:**

	*U*^11^	*U*^22^	*U*^33^	*U*^12^	*U*^13^	*U*^23^
C1	0.057 (3)	0.061 (3)	0.062 (3)	0.007 (3)	0.017 (2)	0.013 (2)
C2	0.055 (3)	0.051 (3)	0.066 (3)	0.006 (2)	0.012 (2)	0.010 (2)
C3	0.047 (2)	0.059 (3)	0.038 (2)	0.008 (2)	−0.0024 (18)	0.0035 (19)
C4	0.054 (3)	0.081 (4)	0.040 (2)	0.013 (3)	−0.001 (2)	0.002 (2)
C5	0.067 (4)	0.115 (6)	0.052 (3)	0.014 (4)	0.020 (3)	0.005 (3)
C6	0.071 (4)	0.103 (5)	0.059 (3)	−0.008 (3)	0.019 (3)	0.020 (3)
C7	0.069 (3)	0.062 (3)	0.056 (3)	−0.009 (3)	0.005 (3)	0.008 (2)
C8	0.040 (2)	0.063 (3)	0.037 (2)	−0.002 (2)	−0.0032 (17)	0.0036 (19)
C10	0.047 (2)	0.054 (3)	0.046 (2)	0.014 (2)	0.0038 (19)	−0.005 (2)
C11	0.063 (3)	0.065 (3)	0.071 (3)	0.008 (3)	0.009 (3)	0.008 (3)
C12	0.100 (5)	0.087 (4)	0.057 (3)	0.035 (4)	0.007 (3)	0.015 (3)
C13	0.072 (4)	0.093 (5)	0.061 (3)	0.030 (4)	−0.017 (3)	−0.022 (3)
C14	0.052 (3)	0.088 (4)	0.079 (4)	−0.002 (3)	−0.006 (3)	−0.019 (3)
C15	0.054 (3)	0.064 (3)	0.065 (3)	0.000 (3)	0.007 (2)	−0.002 (2)
N1	0.049 (2)	0.057 (2)	0.049 (2)	0.0085 (18)	0.0068 (17)	0.0049 (17)
O1	0.090 (3)	0.096 (3)	0.0455 (19)	0.027 (2)	0.0168 (18)	−0.0023 (19)
O2	0.068 (2)	0.062 (2)	0.090 (3)	0.001 (2)	−0.005 (2)	−0.014 (2)
S1	0.0549 (7)	0.0595 (8)	0.0494 (7)	0.0101 (6)	−0.0010 (5)	−0.0080 (5)
C9	0.074 (4)	0.090 (4)	0.050 (3)	0.030 (3)	0.006 (3)	0.002 (3)
Br1	0.0703 (18)	0.118 (3)	0.0437 (13)	0.035 (2)	0.0030 (8)	−0.0089 (11)
Br1A	0.120 (4)	0.059 (2)	0.068 (2)	−0.016 (3)	0.0095 (16)	0.0012 (13)

### Geometric parameters (Å, °)

**Table:**

C1—C2	1.332 (7)	C11—C12	1.381 (4)
C1—N1	1.398 (7)	C11—H11	0.9300
C1—H1	0.9300	C12—C13	1.386 (5)
C2—C3	1.448 (7)	C12—H12	0.9300
C2—H2	0.9300	C13—C14	1.379 (4)
C3—C4	1.392 (7)	C13—H13	0.9300
C3—C8	1.398 (7)	C14—C15	1.382 (4)
C4—C5	1.380 (4)	C14—H14	0.9300
C4—C9	1.505 (8)	C15—H15	0.9300
C5—C6	1.381 (9)	N1—S1	1.661 (4)
C5—H5	0.9300	O1—S1	1.421 (4)
C6—C7	1.387 (9)	O2—S1	1.403 (4)
C6—H6	0.9300	C9—Br1	1.903 (6)
C7—C8	1.379 (7)	C9—Br1A	1.9115 (10)
C7—H7	0.9300	C9—H9A	0.9700
C8—N1	1.402 (6)	C9—H9B	0.9700
C10—C15	1.381 (4)	C9—H9C	0.9700
C10—C11	1.386 (4)	C9—H9D	0.9700
C10—S1	1.749 (4)		
			
C2—C1—N1	110.6 (4)	C12—C13—H13	119.4
C2—C1—H1	124.7	C13—C14—C15	119.4 (5)
N1—C1—H1	124.7	C13—C14—H14	120.3
C1—C2—C3	107.3 (5)	C15—C14—H14	120.3
C1—C2—H2	126.4	C10—C15—C14	118.9 (5)
C3—C2—H2	126.4	C10—C15—H15	120.5
C4—C3—C8	120.7 (5)	C14—C15—H15	120.5
C4—C3—C2	132.0 (5)	C1—N1—C8	107.5 (4)
C8—C3—C2	107.2 (4)	C1—N1—S1	122.8 (4)
C5—C4—C3	117.0 (5)	C8—N1—S1	125.8 (3)
C5—C4—C9	121.2 (5)	O2—S1—O1	120.3 (3)
C3—C4—C9	121.7 (5)	O2—S1—N1	107.0 (2)
C4—C5—C6	121.4 (6)	O1—S1—N1	104.9 (2)
C4—C5—H5	119.3	O2—S1—C10	109.2 (2)
C6—C5—H5	119.3	O1—S1—C10	109.7 (2)
C5—C6—C7	122.6 (6)	N1—S1—C10	104.7 (2)
C5—C6—H6	118.7	C4—C9—Br1	111.1 (4)
C7—C6—H6	118.7	C4—C9—Br1A	111.9 (3)
C8—C7—C6	115.8 (6)	C4—C9—H9A	109.4
C8—C7—H7	122.1	Br1—C9—H9A	109.4
C6—C7—H7	122.1	Br1A—C9—H9A	114.5
C7—C8—C3	122.4 (5)	C4—C9—H9B	109.4
C7—C8—N1	130.3 (5)	Br1—C9—H9B	109.4
C3—C8—N1	107.3 (4)	Br1A—C9—H9B	103.3
C15—C10—C11	122.4 (4)	H9A—C9—H9B	108.0
C15—C10—S1	117.5 (3)	C4—C9—H9C	108.8
C11—C10—S1	120.1 (3)	Br1—C9—H9C	103.5
C12—C11—C10	118.1 (5)	Br1A—C9—H9C	108.9
C12—C11—H11	121.0	H9B—C9—H9C	114.5
C10—C11—H11	121.0	C4—C9—H9D	108.7
C11—C12—C13	120.0 (5)	Br1—C9—H9D	116.2
C11—C12—H12	120.0	Br1A—C9—H9D	110.3
C13—C12—H12	120.0	H9A—C9—H9D	101.4
C14—C13—C12	121.2 (5)	H9C—C9—H9D	108.2
C14—C13—H13	119.4		
			
N1—C1—C2—C3	1.5 (6)	C13—C14—C15—C10	0.2 (9)
C1—C2—C3—C4	−178.7 (5)	C2—C1—N1—C8	−2.3 (6)
C1—C2—C3—C8	−0.2 (5)	C2—C1—N1—S1	−161.2 (4)
C8—C3—C4—C5	−0.4 (7)	C7—C8—N1—C1	−179.0 (5)
C2—C3—C4—C5	178.0 (5)	C3—C8—N1—C1	2.1 (5)
C8—C3—C4—C9	176.4 (4)	C7—C8—N1—S1	−20.9 (7)
C2—C3—C4—C9	−5.2 (7)	C3—C8—N1—S1	160.2 (3)
C3—C4—C5—C6	1.3 (8)	C1—N1—S1—O2	−159.9 (4)
C9—C4—C5—C6	−175.5 (5)	C8—N1—S1—O2	45.2 (4)
C4—C5—C6—C7	−0.2 (10)	C1—N1—S1—O1	−31.1 (5)
C5—C6—C7—C8	−1.6 (9)	C8—N1—S1—O1	174.0 (4)
C6—C7—C8—C3	2.5 (7)	C1—N1—S1—C10	84.4 (4)
C6—C7—C8—N1	−176.3 (5)	C8—N1—S1—C10	−70.6 (4)
C4—C3—C8—C7	−1.5 (7)	C15—C10—S1—O2	173.1 (4)
C2—C3—C8—C7	179.7 (4)	C11—C10—S1—O2	−6.3 (5)
C4—C3—C8—N1	177.5 (4)	C15—C10—S1—O1	39.4 (5)
C2—C3—C8—N1	−1.2 (5)	C11—C10—S1—O1	−140.0 (4)
C15—C10—C11—C12	−0.3 (8)	C15—C10—S1—N1	−72.7 (4)
S1—C10—C11—C12	179.0 (4)	C11—C10—S1—N1	108.0 (4)
C10—C11—C12—C13	−0.1 (9)	C5—C4—C9—Br1	84.3 (6)
C11—C12—C13—C14	0.6 (9)	C3—C4—C9—Br1	−92.4 (6)
C12—C13—C14—C15	−0.6 (9)	C5—C4—C9—Br1A	91.4 (7)
C11—C10—C15—C14	0.3 (8)	C3—C4—C9—Br1A	−85.3 (6)
S1—C10—C15—C14	−179.1 (4)		

### Hydrogen-bond geometry (Å, °)

**Table:**

*D*—H···*A*	*D*—H	H···*A*	*D*···*A*	*D*—H···*A*
C13—H13···Cg1^i^	0.93	2.83	3.716 (6)	160
C9—H9D···Cg1^ii^	0.97	2.92	3.673 (5)	135
C1—H1···Cg2^iii^	0.93	2.69	3.584 (6)	162

Symmetry codes: (i) −*x*+2, −*y*+1, −*z*+1; (ii) −*x*+1, *y*+1/2, −*z*+1/2; (iii) −*x*+2, *y*+1/2, −*z*+1/2.
